# Computer vision distortion correction of scanning probe microscopy images

**DOI:** 10.1038/s41598-017-00765-w

**Published:** 2017-04-06

**Authors:** Iaroslav Gaponenko, Philippe Tückmantel, Benedikt Ziegler, Guillaume Rapin, Manisha Chhikara, Patrycja Paruch

**Affiliations:** 1 Department of Quantum Matter Physics, University of Geneva, 1211 Geneva, Switzerland; 2 Combine Control Systems AB, 413 04 Gothenburg, Sweden

## Abstract

Since its inception, scanning probe microscopy (SPM) has established itself as the tool of choice for probing surfaces and functionalities at the nanoscale. Although recent developments in the instrumentation have greatly improved the metrological aspects of SPM, it is still plagued by the drifts and nonlinearities of the piezoelectric actuators underlying the precise nanoscale motion. In this work, we present an innovative computer-vision-based distortion correction algorithm for offline processing of functional SPM measurements, allowing two images to be directly overlaid with minimal error – thus correlating position with time evolution and local functionality. To demonstrate its versatility, the algorithm is applied to two very different systems. First, we show the tracking of polarisation switching in an epitaxial Pb(Zr_0.2_Ti_0.8_)O_3_ thin film during high-speed continuous scanning under applied tip bias. Thanks to the precise time-location-polarisation correlation we can extract the regions of domain nucleation and track the motion of domain walls until the merging of the latter in avalanche-like events. Secondly, the morphology of surface folds and wrinkles in graphene deposited on a PET substrate is probed as a function of applied strain, allowing the relaxation of individual wrinkles to be tracked.

## Introduction

Atomic force microscopy (AFM), measuring the interaction between a sample surface and the apex of a scanning microscope tip, is an extremely versatile technique which has been successfully applied to the study of materials across multiple domains of scientific research^1^. Not only does this technique make possible three-dimensional imaging of surface morphology with sub-nanometer precision, in metals and insulators, in solid state and biological systems, and in conditions varying from ultrahigh vacuum to full liquid immersion, but an appropriately functionalized AFM tip can also be used as a probe of complex physical properties such as magnetisation, electric polarisation and conductivity, (electro)mechanical responses, adhesion, and friction, all at a local scale^2, 3^. This great versatility allows spatially resolved maps of different functionalities to be built from sequential AFM images, giving a deep understanding of the system under investigation, and the competition and correlation between its mechanical, tribological, magnetic, electric and chemical properties. In materials science, for example, such sequential multifunctional AFM mapping, with combinations of different measurements taken in succession over the same area, has played a crucial role in the discovery of novel functionalities at ferroelectric domain walls^4^, and allowed the microscopic mechanisms of magnetoelectric coupling in complex oxides^5^ to be identified. Not only limited to inorganic materials, AFM techniques have also been used to image dynamic biological processes at the mesoscale, tracking individual molecules^6^ and cell division mechanics^7^.

However, a general problem inherent to all scanning probe microscopy techniques is that two sequentially acquired images are seldom directly comparable, especially down to their (sub)nm limits of resolution. The two main sources of deviation between consecutive images arise from nonlinearities of the piezoelectric actuator used to perform the nanoscale displacements. First, scanner hysteresis, present in all open-loop scanners, will contribute a nonlinear scan-direction-dependent response. Second, varying parameters such as temperature or creep can induce a time-dependent drift which will shift the images with respect to each other. Various techniques have been developed in order to combat these sources of error. Continued improvements of the mechanical stability, as well as the introduction of closed loop scanners^8^ - allowing the precise position of the tip with respect to the sample to be determined - have greatly simplified the problem of nonlinearities and drift. In systems where such hardware improvements were not possible, software algorithms have been introduced for an online correction of the tip positioning, taking into account the nature of the piezoelectric tube scanner - with the requirement of complex calibration and limited success in real-world applications^9, 10^. Finally, offline software correction algorithms applied directly to the acquired images were developed, mapping topographic features detected during sequential scans onto each other to correct for thermal drift induced position shifts during the actual scanning process^11, 12^.

In this paper, we present an approach in this last category, a software algorithm for offline correction of atomic force microscopy images. The fully automated computer vision enhanced algorithm was designed in order to tackle both the scanner nonlinearities as well as thermal drifts with two goals in mind. First, since complete mapping of different functionalities at the nanoscale usually requires multiple AFM scans over the same region, a cross-correction of different data types is necessary to extract the functional behaviour precisely at each position on the surface. This is the case for instance of successive piezoresponse force microscopy (PFM) and conductive tip AFM (CAFM) imaging. Secondly, the imaging of dynamical processes is associated with multiple scans over the same area, resulting in images with overlapping portions that, once corrected, allow time- or stimulus-resolved functionality maps to be constructed. The innovative use of computer vision libraries allows for optimal topographic feature detection and mapping both for a precision tracking of minute topographic changes, as well as for the cross-correction of secondary image channels. After discussing the validity and implementation of the algorithm we present its application to two systems demonstrating our goal-oriented design. The cross-correction is shown by the mapping of ferroelectric polarisation switching as a function of time, demonstrating the robustness of our approach for space-time-correlated imaging of local functionalities. A second example tracks the response of wrinkles in chemical vapour deposition graphene as a function of applied strain, showing their progressive relaxation.

## Distortion correction algorithm

**Figure 1:**
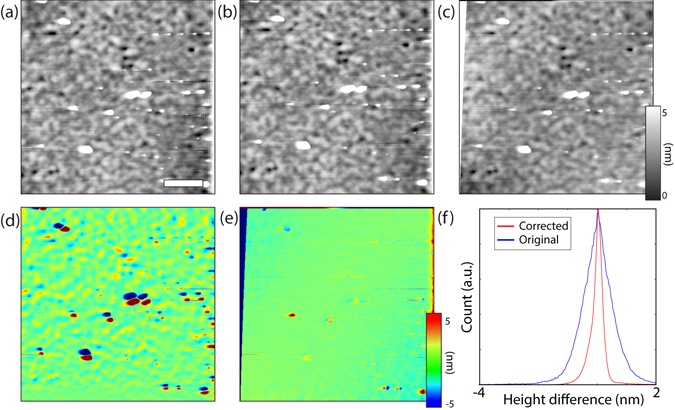
(**a**,**b**) Subsequent topographic images acquired on a PZT thin film, with the white scale bar representing 1 *μ*m. (**d**) The height difference between the uncorrected images demonstrates that the two images cannot be directly correlated spatially. The corrected image (**c**), however, shows a substantially lower variation in the height difference (**e**), demonstrating the effectiveness of the algorithm. This is further substantiated by the collapse of the histogram of height differences (**f**), with the corrected histogram having a narrower distribution around 0 nm.

A concrete example of the necessity of a reliable correction method is demonstrated in Fig. 1. Two sequentially acquired topographic images of a Pb(Zr_0.2_Ti_0.8_)O_3_ (PZT) thin film in Fig. 1(a,b) look identical to the eye. However, their differential height map, seen in Fig. 1(d) clearly shows that this is not the case - the particles in the first image (black) not aligning with particles in the second image (white). Thus, automated tracking of functionality at the nanoscale is precluded by the drifts and distortions. However, when the topography of the second image is corrected with the method described in this paper, as can be seen in Fig. 1(c), the corresponding differential height map in Fig. 1(e) is almost featureless, demonstrating the effectiveness of the correction. A normalised histogram of the uncorrected and corrected height difference maps is shown in Fig. 1(f), in blue and red respectively, indicating a much closer matching between the two images for the corrected version of the topography.

### Correction validity

In order to demonstrate the validity of the corrections for the two main noise sources, some assumptions are necessary. First, we postulate that drift and hysteresis act independently on the fast and slow scan directions (no cross-correlation), and thus any contribution can be split into orthogonal components, parallel and perpendicular to the scan direction. A second assumption is that the drift is negligible within a single scan frame, given that once the scanner has stabilized the average time dependent drifts are of the order of nanometers per minute - small enough to not have major effects in a typical 10–20 minute measurement over a region of 1–5 micrometers. Thus, we can relate the drift between two consecutive images as a linear (*x*, *y*) position shift between two images. This leaves us with the second source of deviation - scanner nonlinearities such as hysteresis - whose contribution is nontrivial due to the physical properties of piezoelectric materials. Nevertheless, models have been developed in order to study this hysteresis, allowing for a precise description of the relative displacement error as a function of position, yielding an effective position *x*_*t/r*_ as a function of the control voltage *V* that can be expressed as ${x}_{t/r}=bV\mp \frac{u}{\alpha }(1-\frac{2}{{e}^{\mp \alpha {V}_{{\rm{\max }}}}+{e}^{\mp \alpha {V}_{{\rm{\min }}}}}{e}^{\mp \alpha V})$ ^13^. The *t* and *r* branches of the equation describe the trace and retrace behaviour respectively, and *α*, *b*, *u* are constants. For the correction of the hysteresis, we assume that the images were acquired consecutively, with the first image being an up-directed scan followed by a down-directed scan (or vice versa), although the computation is valid for any two images of the same area performed under identical measurement conditions (speed, size, number of pixels) regardless of their consecutiveness. This is due to the fact that two images acquired in the same direction will simply have a negligible error due to hysteresis, whereas two images that have one of the two (slow or fast) scan axes inverted will be handled correctly by the algorithm. Thus, the difference in effective position as a function of voltage for the two images is given by ${\rm{\Delta }}x(V)={x}_{r}(V)-{x}_{t}(V)=-\frac{2u}{\alpha }$ $(\cosh (\frac{1}{2}\alpha (-{V}_{max}-{V}_{min}+2V))/\cosh (\frac{1}{2}a({V}_{max}-{V}_{min}))-1)$. This expression is consistent with observations, as the minimum error is found at the edges of the scan, ${\rm{\Delta }}x({V}_{{\rm{\min }}})={\rm{\Delta }}x({V}_{{\rm{\max }}})=0$, and the maximum is found in the middle of the image, at $V=\frac{{V}_{{\rm{\max }}}+{V}_{{\rm{\min }}}}{2}$ - as expected. The expression for Δ*x*(*V*) can be reformulated in terms of a polynomial expansion of order 2, with the deviation from the original expression of less than 1% for the typical values of voltage used in conventional piezoelectric scanners. Thus, adding up the drift and nonlinearities, the two sources of error can be approximated by a second order polynomial - demonstrating the soundness of the method for the correction of images. With the above reasoning, correction turns into the the sequential problem of locating a large number of distinctive features in a comparable data set for both images, extracting their relative displacement, and computing a position correction function which will then be applied to the data set to be corrected. The correction can thus be generated for any two data sets, and can be used for either the tracking of the area of interest over multiple images or for cross-correction of a different data set acquired simultaneously, such as the current that is acquired at the same time as the topography in CAFM measurements.

### Implementation

**Figure 2:**
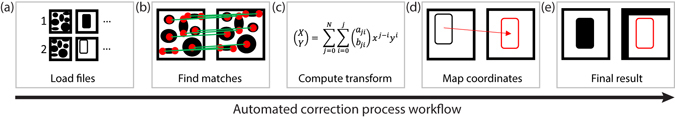
Illustration of the algorithm workflow. Images are loaded with a *Python* script (**a**), and the topographic channels are analysed through a computer vision algorithm in order to find a complete set of matching points (**b**). The matching points are then used to generate a coordinate transform (**c**,**d**) which is finally applied to the auxiliary data channels (**e**), enabling nanoscale correlations to be extracted.

The workflow of the algorithm is described in Fig. 2. First, the two images to be compared are loaded by the use of a custom AFM image loading module that accepts standard measurement file formats. Then, the reference channels - in most cases topographies - are extracted from both images, and processed through the *OpenCV* computer vision library in order to find pairs of matching key-points between the two images, yielding an array of points with their respective (*x*, *y*) coordinates in both images. The initial detection of features is performed by the use of off-the-shelf methods relying on the multi-scale analysis of local variations which allow to extract unique features such as edges, corners or objects. In our case, feature detection with the *SURF* (*Speeded-Up Robust Features*)^14^ and *ORB* (*Oriented FAST and Rotated BRIEF*)^15^ algorithms has given the best results during testing. The resulting features are then analyzed between both images by a *FLANN* (*Fast Library for Approximate Nearest Neighbours*) matcher^16^, designed for nearest neighbor search, generating a complete set of matching pairs of points between the first and second measurement. These two sets of positions are then used in order to find a polynomial transform of the coordinates between the first and second images by means of the *scikit-image* transformations module. This transform is finally applied onto the auxiliary channels such as the phase and the current through the use of the coordinate mapping function of the *scipy* n-dimensional image module in order to generate a final corrected image. The complete implementation in *Python* can be found in the Supplementary Materials as well as on the *GitHub* repository provided^17^.

## Results and Discussion

In order to demonstrate the viability of the distortion correction algorithm in real-world applications, two representative examples of the analysis of scanning probe microscopy images are shown. First, the nanoscale time-dependence of voltage-induced ferroelectric polarisation reversal is explored in a series of piezoresponse force microscopy images, from which we extract the dynamics of ferroelectric domain nucleation, growth and merging. Second, the relaxation of wrinkles in chemical-vapour-deposition (CVD)-grown graphene on a polyethylene terephthalate (PET) substrate under progressive bending strain is followed, allowing morphological changes to be tracked as a function of strain. In both cases, the distortion correction algorithm is used to gain quantitative insight into fundamental processes that were previously accessible at best qualitatively. Without such a correction, in the ferroelectric films we could image overall switching behaviour, but not track domains individually, and in the graphene samples, while we could observe that in general wrinkles appeared to decrease in size and in some cases disappear when strain is applied, we could not directly compare these changes in individual wrinkles as a function of position and orientation across the entire image.

### Nanoscale polarisation reversal tracking

Ferroelectric materials such as PZT possess a spontaneous electric polarisation, organising into regions with different orientation, known as domains, separated by thin interfaces known as domain walls. The ferroelectric polarisation can be reversed by an electric field applied to the material either with planar electrodes in a capacitor geometry, or by means of a biased scanning probe microscopy tip. In the former case, the macroscopic properties of the material can be measured, but the microscopic behaviour of individual polarisation switching events is lost in the response^18, 19^. Thus, atomic force microscopy has become over the last decades the tool of choice for probing ferroelectricity at the nanoscale by means of local polarisation switching and piezoresponse force microscopy (PFM) imaging, allowing the non-linear dynamics and characteristic roughness scaling of domain walls, and their interaction with defects to be directly observed^20, 21^. Moreover, recent studies have shown the domain walls themselves to possess a plethora of localised functional properties absent from the surrounding parent phase, making them potentially useful as active device components of a future domain-wall-based nanoelectronics^4, 22, 23^. In such studies, in order to correlate the emergence of complex behaviour and the position of the domain walls, a wide variety of AFM techniques is used in conjunction with PFM - most of them requiring multiple scans of the same regions in order to both image the domain structure and probe the functional response. These, and even more simple time series with consecutive PFM scans of the same domain structure, usually suffer from the problems described in the previous sections, requiring careful human inspection to compare the sequential measurements - a problem that disappears with the advent of smart data correction algorithms such as the one described in this work.

**Figure 3:**
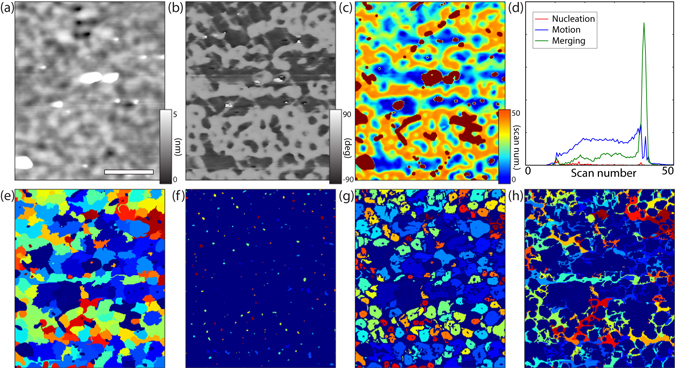
Investigations of the domain structure of a PZT thin film whose topography is shown in (**a**). The domain structure at a given time point (**b**) is noisy, so an in-depth analysis is used to extract the time-of-switching map (**c**). The map can be analysed and broken down into domain nucleation (red), motion (blue), and merging (green) events whose time distribution is shown in (**d**). Each domain can then be individually tracked from its nucleation onward, illustrated in one colour per domain in (**e**). The domain nucleation, motion and merging regions are shown in (**f**–**h**) respectively, opening the pathway to an analysis of the defect landscape and domain avalanche behaviour.

In order to illustrate the usefulness of our algorithm for the study of ferroelectrics, we look at domain switching dynamics in a 100 nm thick PZT *c*-axis oriented thin film epitaxially grown on a conductive Nb:SrTiO_3_ single crystal substrate by radio frequency magnetron sputtering. The film is mono-domain, up-polarised as grown, and exhibits a slightly granular morphology as shown in Fig. 3(a), with small particles (bright contrast in the height image) on the surface. The measurements were performed by using dual frequency resonance tracking PFM on a *Veeco Multimode IV* microscope equipped with a *Nanonis* 4 controller. When the sample is scanned continuously in sequential 90-second 5 *μ*m^2^ 512 × 512 pixel scans while a positive 10 V bias is applied to the AFM tip, a complex domain structure emerges as a result of multiple nucleation and growth events - with an example of the polarisation at the 30th scan (after 45 minutes of scanning) shown in Fig. 3(b). Small domains nucleate in the up-polarised background, grow radially outwards, and merge until the whole scanned area switches to the uniformly down-polarized state, with the exception of a few regions apparently frozen in the up-polarised state, or where the presence of significant surface particles makes both polarisation switching and imaging difficult.

We note that in this measurements series, the biased AFM tip spends very little time over a given region during each scan, due to the high scan speed used. Thus, although the applied voltage is well over the minimum value of 5 V for which stable polarisation reversal is observed under a stationary tip in PZT thin films^24^, at each pass in the sequence this voltage is effectively applied for a duration of only ≈300 μs per pixel. The applied voltage being 10 V, by proximity effect nearby pixels will also feel a super-coercive field, thus effectively bringing the “exposure” of each point of the sample to 5–10 ms. As previously reported^25^, for such short times the polarisation under the tip is not fully reversed, but instead a potentiated region forms, which can be gradually driven to full reversal by multiple short duration voltage pulses. Thus, this high speed dynamic measurement allows us to qualitatively probe domain nucleation and growth - and to understand the way domain walls explore the defect landscape around them.

For the analysis, fifty consecutive scans were selected, with a cut-off chosen at the scan by which the polarisation had acquired its final configuration (75 minutes). The images were aligned by using the distortion correction algorithm with the vertical deflection channel, using the 20th scan (30 minutes) as the reference with respect to which all others were corrected. The quality of the correction is demonstrated in the movies available as part of the Supplementary Materials. Once the correction was applied, each pixel of the image could be tracked over the full experimental duration of 50 scans until the final reversed polarisation configuration. The evolution of the latter at each pixel was then extracted and subjected to a Heaviside step function fit to identify the time at which its polarisation state was reversed from up to down - with a corresponding shift of 180° in the piezoresponse phase channel. The step function fits yielded an effective time-of-switching for each pixel, as shown in Fig. 3(c). The black spots represent areas that had not switched and that were thus excluded from further analysis. Using this map, it was possible to track each domain from its nucleation, through its outward growth and up to its merging with the domains around it. To categorise the appearance of switched regions in successive images, these were separated by the following criteria: if the new region does not have neighbouring domains within a radius of one pixel around it, it is considered a nucleation event; when it has a single neighbouring domain, then it is considered a motion event associated with that domain; and finally when there is more than one neighbouring domain, then it is considered a merging event, and the region is associated with the domain which has the most neighbouring pixels. We could thus extract the complete domain map shown in Fig. 3(e), as well as mapping the three types of switching event: nucleation in Fig. 3(f), domain wall motion in Fig. 3(g) and merging in Fig. 3(h). Moreover, by extracting the time-of-switching for each of these maps, we could separate the polarisation switching events into the three categories as a function of time in Fig. 3(d).

The observed switching behaviour is in very good agreement with the qualitative understanding of ferroelectric polarisation several based on both experimental and theoretical studies^21, 26–29^. From previous observations in thin films, polarisation reversal usually happens first by means of nucleation at sites with a lower activation potential related to the complex defect landscape of the material^30–33^. Once nucleation occurs, the resulting domains grow outwards, with characteristic dynamics dominated by domain wall motion through a strongly varying potential energy landscape^21, 34^. Finally, when two growing domains get close enough to each other, they can merge into a single domain, minimising the domain wall length and thus energy cost of the resulting polarization configuration. This behaviour is visible in the results presented in Fig. 3, with the nucleation sites distributed randomly around the scanned area, shown in Fig. 3(f). The resulting domain grow radially around the nucleation sites, as indicated in Fig. 3(g). As the domains get closer and closer together, they appear to snap into one another within the time interval of a single scan, generating avalanche-like merging events as shown in Fig. 3(h), previously reported in magnetic domains^35^, and here observed for the first time directly in ferroelectrics. This sequence of switching events appears to be a completely general behaviour, as can be seen from the timeline of the various types of events, shown in Fig. 3(d). Nucleation events, shown in red, occur at specific instants of time, and are immediately followed by an increase in motion events (blue). Merging events (green) become apparent only later, with a very large peak observed before all three event counts decay due to quasi-complete switching.

This short study makes the usefulness of our distortion correction algorithm for the investigation of ferroelectric domain dynamics under applied field very clear. Not only is it possible to track the evolution of individual domains, but also to separate the nucleation, motion, and merging events which collectively contribute to polarisation reversal, enabling in-depth nanoscale data analytics to be performed on the time- and voltage-dependent switching behaviour of ferroelectric thin films. This can allow a microscopic understanding of the processes involved in the domain wall motion dynamics and their interplay with defects, paving the way to better control of macroscopic functionality.

### Wrinkle relaxation in strained graphene

Although the cross-channel correlation presented above is the major strength of the distortion correction algorithm, a direct tracking of topographic changes can also be useful for morphological studies of surface-mediated processes. Examples of such a use include the dynamics of molecules in response to stimuli^36^, and the atomic tracking of single defect dynamics in scanning tunnelling microscopy^37^. Here, as an example, we present the relaxation of wrinkles in a 1 cm^2^ CVD graphene layer deposited on a polyethylene terephthalate substrate in response to bending strain.

**Figure 4:**
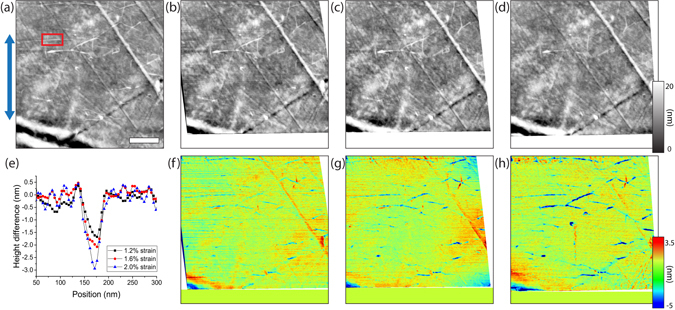
CVD graphene on polyethylene terephthalate imaged at different tensile strains. The AFM topographies (**a**–**d**) at nominal strains of 0%, 1.2%, 1.6% and 2%, respectively, show the progressive disappearance of wrinkles as the bending strain is increased in the direction of the blue arrow. Thanks to the distortion correction, the differential height images (**f**–**h**) between the height images at 0% and 1.2%, 1.6% and 2% strain, respectively, demonstrate the disappearance (blue features) of wrinkles perpendicular to the strain direction (red arrow) whereas the remaining area shows little height difference (green-yellow region). The average height differential of the wrinkle highlighted in (**a**) is shown in (**e**), demonstrating a decrease in height as a function of strain.

The graphene was strained *in-situ* using a home-built device based on a micrometer gauge, shown in, designed to allow both optical and scanning probe microscopy measurements of the system under applied strain, as reported in ref. 38. The scanning probe imaging in this study was performed on a *Veeco Dimension* 3100 AFM with a *Hybrid* XYZ closed-loop head. The closed-loop scanner facilitated the analysis, as the hysteresis due to the piezo-column was directly compensated. The topographic imaging shown in Fig. 4(a–d) was performed in intermittent contact mode on 5 *μ*m^2^ areas at 0%, 1.2%, 1.5% and 2% nominal strain, respectively, with the tip retracted between each measurement in order to readjust the strain.

These topographic images in Fig. 4(a–d) are all of high quality, and show very similar morphology across the different nominal strain conditions, with a root-mean-square surface roughness of ≈3 nm, revealing a clean graphene surface almost free from PMMA residues, and characterised by wrinkles and folds of different heights and orientations. A closer inspection reveals that as strain is increased along the direction indicated by the blue arrow in Fig. 4(a), there is a progressive height decrease and disappearance of wrinkles perpendicular to the strain direction. Although this observation can be obtained by qualitative visual inspection, the different drifts of the system preclude a systematic quantitative analysis. However, thanks to the distortion correction algorithm, the height difference maps in Fig. 4(f–h) can be generated, clearly showing the positions at which the wrinkles appear (red) and disappear (blue), parallel and perpendicular to the applied strain direction respectively. The average height differential of the wrinkle highlighted in Fig. 4(a) is shown in Fig. 4(e) as a function of strain. Such wrinkle relaxation in fact means that the effective strain *ε* applied to the graphene is smaller than the nominal values. In a more detailed study of the effects of strain application on the optical conductivity of graphene in the far infrared regime^38^, additional Raman measurements were therefore used to calibrate effective vs. nominal strain, by comparison to a mechanically exfoliated single graphene flake^39^.

Thanks to the distortion correction method, the comparison between the topographies at different strain values can be used in order to study the effect of wrinkle relaxation *in-situ* as a way to relieve strain, an important consideration for graphene based sensors and electronics in strain-resilient device applications.

## Conclusion

We have presented an automated distortion correction algorithm based on computer vision, and demonstrated the rationale behind its applicability to the correction of the nonlinearities of piezoelectric column nano-positioners. The method, based on the mapping of coordinate transforms between matching sets of points between two images, enables offline processing and complex data analytics of preexisting images. Different transforms were described, with the polynomial transform treated as the generic one-fits-all solution. The algorithm was then applied to two distinct systems both to demonstrate the power of cross-correlation and of topographic feature tracking. Whether it is the intricacies of domain nucleation, growth and merging in PZT thin films or the relaxation of wrinkles on strained CVD graphene, signatures of fundamental processes have emerged - allowing for a better comprehension of the physics underlying the studied systems.

Moreover, the methods demonstrated above are not limited to scanning probe micrographs, but can also be generalised to any sequence of images where a large enough set of matching features can be detected. This is the case of optical microscopy images - whose distortion can be mapped to secondary acquisition channels such as fluorescence, in order to study biological processes. Another field which could benefit from this offline correction is high resolution transmission electron microscopy, where time-resolved spatial analysis can provide insight into fundamental materials physics. Overall, the computer vision based approach to offline image sequence correction is a versatile tool for a broad community of microscopists, opening paths towards a greater understanding of materials systems.

## Electronic supplementary material

Dataset 1
